# The Nursing Supplement

**Published:** 1888-06-09

**Authors:** 


### The Nursing Mirror.

All Contributions for this Supplement should be addressed "The Nursing Mirror," care of The Editor, 38, Old Jewry, E.C.

### En Passant.

WORKHOUSE INFIRMARY NURSING ASSOCIATION.—About sixty nurses belonging to the above Association spent a pleasant afternoon in May at the house of Lady Wantage. Tea was provided for all, and then, after a distribution of gratuities and medals, Miss Twining and Miss Wood addressed the meeting. Perhaps the greatest treat of the evening, however, was the singing of Lady Simeon, which greatly delighted the nurses.

THE "SUNDAY AT HOME."—The June number of this magazine contains the first of a series of papers by Mrs. Brewer, on "Hospital Nurses in London and on the Continent." These papers should be interesting, especially when they reach the description of our sister nurses in France and Germany, of whom we unluckily know so little. This first chapter is chiefly introductory, dealing with the training of nurses. Mrs. Brewer says that all is not perfect yet in the nursing world, but that the evils arising from education, refinement, and self-mastery, are infinitely less disastrous to the patients than those arising from ignorance and want of self-control.

AMATEUR WORK.—A good story is just now being circulated. If the original narrator is to be believed, a member of the Ladies' Ambulance Corps was walking along the Strand a few days ago, when she saw a man knocked down by a passing cab. As the poor fellow's leg was said to be fractured, a young woman stepped from the gathering crowd and at once volunteered to put the limb in splints. In the absence of proper materials, she borrowed from the sympathising onlookers a walking-stick, a parasol, and about a dozen handkerchiefs. Thus furnished, she dexterously applied her material, and was rewarded for a skilful undertaking by the warm praises of those around her. On proceeding to raise the sufferer, however, it was discovered that matters were not quite right. An investigation revealed the fact that the wrong leg had been set, and (says the London Corespondent of the *Newcastle Chronicle*) the bandages, as a consequence, were sadly and slowly unwound.

A BRAVE HOSPITAL NURSE.—The following paragraph has been going the round of the papers : "A few days since a child aged two years was removed by order of Dr. Watson, medical officer to Lieutenant Williams's Infectious Hospital, Rochester, to hospital, suffering from a bad form of diphtheria. Upon the child's arrival it was found that the symptoms were of a virulent character, necessitating an immediate resort to tracheotomy. During the night the tube became clogged, and the poor little sufferer was found gasping for breath. Death being imminent, one of the senior nurses, a young woman named Finns, bravely offered to suck the tube, a task attended with almost certain risk of her life. Notwithstanding urgent representations made to her as to the danger attending such self-sacrificing conduct, Nurse Finnis applied her lips to the tube, and thus prolonged the child's life for several hours. This heroic act has excited warm admiration, and the medical staff propose to represent the case in the proper quarter." We are quite sure Nurse Finns would rather have had the last sentence omitted; such a deed can never be rewarded save by the great wave of sympathy and loving esteem which must arise in every heart, as they hear of one more act of absolute unselfishness. It is the record of such heroic deeds which keeps bright our enthusiasm through years of drudgery, and maintains through all disappointments our faith in humanity.

NEWS FROM WORCESTER.—We are sorry to hear that, after five years of hard work, the matron of Worcester Infirmary has resigned. Miss Gwyn feels the need for a rest, and in retiring she can find satisfaction in the knowledge that she leaves the infirmary in a much better state than she found it, though her predecessor, Miss Sutcliffe (now of the Women's Hospital Soho), did a great deal during the short time she was matron there. Worcester Infirmary contains 111 beds, of which an average of 87 are occupied each day. The number of out-patients last year was over four thousand. This makes a good school for nursing, and several probationers are trained yearly. The infirmary has lately been enlarged and the kitchen and offices improved, so that whoever is lucky enough to secure the post vacated by Miss Gwyn, will find all in perfect working order.

WOMEN ORATORS.—It is becoming quite common for women to speak publicly on those subjects with which they are well acquainted, especially at meetings connected with nursing matters. Mrs. Stuart Wortley has frequently addressed a large audience on behalf of district nursing, and Lady Burdett-Coutts made quite a long speech at the annual meeting of the Grosvenor Hospital for Women and Children. She said, amongst other things, that the warmest, kindest, and most untiring nurses were not always the best or most capable, and it was not infrequently the case that patients would rather have persons about them who were not so intimately and particularly attached to them. Many a patient had suppressed feelings of discomfort, anxiety, or pain even, wishing that those around tenderly and anxiously watching might not know their sufferings, and a good and Christian nurse was in all houses a very great comfort and boon. Of course there were women in all ranks of life who were born nurses, who could supply all that the best nurse could give, and a good example was to be found in that Imperial lady who was the object of so much of their sympathy, and whose untiring care and undaunted courage would endear her name both to the English nation and to the nation of which she had become a countrywoman. Many people will agree with the Baroness that in sickness a stranger is often the most pleasant nurse ; and surely all nurses will agree with the opposite proposition that a stranger is always the most pleasant patient.

THE MIDWIVES' INSTITUTE.—On June 1st the annual meeting of the Midwives' Institute and Trained Nurses' Club was held at 15, Buckingham Street. The treasurer reported on the finances of the society, which are not very flourishing, and might be improved by an increase of members. A good number of nurses have joined since the last report was read, but many have never even heard of the club and the advantages it offers. It now has a splendid medical library, and the table is strewn with weekly papers, including the *British Medical Journal*, *Lancet*, &c. The lectures are also most interesting and instructive. With regard to the proper registration and licensing of midwives there was much discussion, and the absence of any law on the matter was deplored. England is the only civilised country where any woman who likes can put "Midwife" on her door, and then practice. The Institute has done good work by only admitting to its membership those who possess the diploma of the Obstetrical Society, and by endeavouring to enlighten the public as to the difference between a midwife and a monthly nurse. A midwife is able to attend confinements without a doctor, and therefore it can easily be understood that she must be thoroughly trained, as on her promptitude the lives of mother and child often depend. Some members proposed that the Institute should hold drawing-room meetings, and seek Royal patronage. It ought to be able to hold its own without that if those whom it benefits would support it properly, and if more members were forthcoming.

### The National Pension Fund for Nurses.

By Dr. J. S. Bristowe, F.R.S., Senior Physician to St. Thomas's Hospital, and President of The Hospitals Association, being the continuation of his Address from p. 170.

The Hospitals Association must naturally take a lively interest in the National Pension Fund for Nurses; for although the Association, as an association, has no voice in the management of the Pension Fund, it was at the meeting of the Association in October last that publicity was first given to the fact that such a society was about to be established, and that the main conditions and objects of the society were explained in a paper read by Mr. Burdett. It was incorporated in February of the present year, and is now at work.

The sole object of the managers of the Fund is to benefit nurses: firstly, by providing them with the means, in return for certain annual subscriptions, of securing certain annuities commencing from certain ages; and, secondly, by enabling them, in virtue of certain subscriptions to a sick fund, to secure certain weekly payments during periods of incapacity from sickness. If the Pension Fund had been an assurance company, founded on ordinary commercial principles, it would have been floated by capital subscribed by shareholders, who would have been entitled to participate in the profits of the undertaking. But the successful institution of this company is due to the munificence of four gentlemen, who between them have deposited with the Accountant-General in Chancery the sum of £20,000 as security for the annuitants and policyholders; and who not only do not exact interest, but have consented that the dividends shall for a time be paid to the Bonus Fund. Mr. J. Morgan, one of the gentlemen above referred to, has given a further sum of £5,000 to be devoted without conditions to the purposes of the Fund. Again, if the Pension Fund were an ordinary assurance company, the directors or council would receive payment for their services, and paid agencies would probably be established in all the chief nursing centres. It is obvious, therefore, in the case of the Pension Fund, that most of the ordinary sources of expenditure which hamper assurance companies have been avoided; that the actual annual expenditure must be relatively very small; and that the tax upon the policyholders' premiums thus saved will go sooner or later, but certainly, to the policyholders, either in diminution of their annual payments, or in enhancement of their pensions. It must be admitted that even so the annual payments of nurses, especially of such nurses as begin to subscribe late in life, must appear large, and in many cases may (if unaided) be prohibitive. But unfortunately facts are inexorable, and no juggling with figures can make it possible to ensure for a subscriber a larger pension than a due proportion between the amount of money she has paid up to the age when her annuity begins, and the estimated duration of her life after that date, entitles her to receive.

The necessarily heavy annual rate of payment which subscription to a self-sustaining assurance company entails was not overlooked by those who founded the National Pension Fund. But it was felt by them that not only is nursing an occupation which demands the highest qualities of womanhood, but that from the nature of their work nurses must at best be content with scanty incomes, and in many cases work at starvation wages, or from the simple motive of self-sacrificing charity; and on these grounds it was proposed, and it is hoped, that the subscriptions of the nurses themselves will be supplemented by persons of means, and by institutions employing nurses, and that a bonus fund (additional to that accruing from the nurses' own subscriptions) will thus be created in substantial mitigation of the apparently hard terms of the actual contract which, on grounds of safety to the nurses themselves and on strict business principles, the company is compelled to offer.

The press generally has fully recognised the purity of the motives of those who founded and those who are managing the Pension Fund, and have expressed approval of their aims and action. But there is one journal, the *Lancet*, which professes to think differently. Now, no one connected with the Pension Fund has any right to complain of the honest adverse criticism of competent persons who have taken the trouble to investigate the whole subject for themselves and to understand it. The subject is a difficult one, and one concerning certain details of which it is not improbable that even skilled actuaries might be divided in opinion. And such criticism might quite possibly point out defects in the scheme which had been overlooked, and thus lead to its material improvement. The criticism of the *Lancet* is not of this kind. The editors have entrusted the subject of the National Pension Fund to be dealt with by a writer who knows nothing about life assurance, and who week by week for some time past has been allowed to disport himself in the editorial columns. I wish it were possible to believe that this delegation of an important matter to incompetent hands was merely an editorial oversight. But, unfortunately for this hypothesis, the *Lancet* has so far identified itself with the writer in question that it has refused to admit into its columns any reply from the actuary to the Fund, and has reprinted the articles relating to the subject in pamphlet form, and distributed them in batches to hospitals and other institutions connected with nursing. I believe that any of my audience can obtain copies by application at the *Lancet* office.

It would be impossible in the time at my disposal this afternoon to discuss these documents point by point, nor would it be worth while. I will, however, with your permission comment briefly on some of the more important charges made or suggested in them.

In the first article an attack is made upon the eleemosynary element in the scheme of the Nurses' Fund, and a letter is quoted with approval, which was sent to the *Times* by certain official members of three women's benefit societies. The writer enlarges upon this subject in a subsequent article. I do not propose to enter upon a discussion of what is meant by charity, who are suitable recipients of charity, and whether charity benefits or demoralises. The subject is one which lends itself to academic discussion, and the writer handles it with characteristic malevolence towards the Pension Fund. But it is obvious that the so-called charitable element constitutes an essential part of the scheme. The pecuniary advances of Lord Rothschild, and Messrs. Gibbs, Hambro, and Morgan, are purely charitable; the gratuitous services of the Council are charitable, and in this respect are in precisely the same category as the contributions which are invited from the general public. Nothing, indeed, is more certain than that the four gentlemen just named, and every one else connected with the establishment and management of the Fund, have from the beginning fully recognised that, in order to be of benefit to nurses, and successful, the Fund must be in part charitable, and that if the charitable element be eliminated it must fail. For few women can afford the annual payments required to insure an adequate income in old age; and of those who can afford them few are likely to be tempted to subscribe a relatively large proportion of their income for the apparently remote and comparatively small returns which would be the equitable and true equivalent of their yearly contributions. To condemn the charitable element is to condemn the Pension Fund. And for those, nurses or others, who condemn it there is nothing for it but to stand aloof. But in this matter I would recommend nurses to deliver their consciences into the hands of the eminent financial authorities and philanthropists above named, rather than to follow blindly the cheap advice of irresponsible moralists.

In the next place he complains that the "Pension tables appear to have been computed on a not very liberal scale," and proceeds in successive letters, and as new arguments suggest themselves, to prove to his own satisfaction the incompetence of the actuary, the dishonesty of the governing body, and the stupidity of all concerned. He bases his charge of illiberality upon the fact that in some two or three companies, whose prospectuses are before him, the rates of premium are lower than those offered by the Pension Fund, and on the assumption that nurses may become deferred annuitants on easier terms in what he calls the open market than in the Pension Fund. There is *prima facie* evidence in favour of his opinion, but if he had pushed his inquiries a little below the surface, or had had any practical acquaintance with life assurance, he would have known that this evidence on which he relies is worthless. There is only one company, namely, the Marine and General, which largely transacts this class of annuity business. This has more of such business than all the other companies put together; but it is confined to mariners, an annuity is never granted, I believe, except in connexion with a life policy, and this company, therefore, may be left out of account. Very few of the other companies court annuity business, even of the immediate (non-deferred) class; many discourage or decline it; and scarcely any, even of those which grant immediate annuities, have a single deferred annuity contract on their books. The fact is that annuity business, even of the immediate class, is not found, on the whole, to be remunerative, and is not therefore sought after as a portion of assurance companies business.

But however that may be, it was the clear duty of the Pension Fund to those whom it invites to invest their money with it, so to frame its tables and conduct its business as to provide in the first place for the certain fulfilment of its contracts, and then to ensure the highest possible returns for the subscriptions deposited with it. Now how was this object to be attained? Certainly not by adopting blindly the lower rates of certain other offices, which were framed with other objects. Still less by rule of thumb, as the *Lancet* would have it. The only legitimate way was to have the matter calculated afresh from the best existing data by a competent actuary and mathematician. It was submitted, therefore, to Mr. King (who is both), with results which have excited the *Lancet's* indignation. He has determined that in order to secure certain rates of pensions for those subscribing for them, the annual premiums cannot, with safety to the assurers, be made smaller than his printed tables make them. The *Lancet* has not endeavoured to prove him wrong either in his data, in his calculations, or in his conclusions. It has not ventured to submit his tables to any competent authority; or if it has, it has prudently abstained from publishing his opinion on them. But it compares his tables with those of two or three other companies; and, without seeking an explanation of the discrepancies it discovers, accuses him of error where he differs from them. Having uttered anathemas against the bonus fund, which it is hoped that the contributions of the charitable will create for the enhancement of the pensions, it accuses the managers and him of dishonesty, because this has not been taken into account for the reduction of the premiums; and having after a while discovered (what if it had taken the least trouble or possessed the slightest knowledge on the subject, it should have known) that women who have reached a certain age have the expectation of a longer life than men who have attained the same age, and that this fact had been taken into consideration in the framing of annuity tables for nurses, who practically are nearly all women, it parades the fact as a fresh proof of wickedness or imbecility.

Now, ladies and gentlemen, I have taken the precaution to do what the editors of the *Lancet* have not done. I have placed all the documents, including the republished *Lancet* articles, in the hands of an actuary of the highest eminence, asking him simply to give me his best judgment upon them; and I need scarcely, perhaps, tell you that he regards the *Lancet's* comments as the merest trivialities, and unworthy of serious notice, and sanctions with hearty approval the data and mode of calculation of Mr. King, and the rates which the Pension Fund has adopted. He points out, what Mr. King himself allows, that the best obtainable data for calculation are not wholly trustworthy; that, as it was of paramount importance that the security of the Fund should be beyond suspicion, it was absolutely necessary that in any case of doubt the bias should be towards increasing the premiums, rather than towards their diminution; that with the data, which were alone at present available, no other rates save those adopted were feasible; and that hence it would not improbably appear in the course of time that the rates at present demanded are really higher than is necessary; but that to have made such reductions at the present time, and without experience of the working of the scheme, would have been a piece of reckless improvidence, and indefensible as a matter of business.

The writer in the *Lancet*, who condemns the charitable element in the Fund, actually complains, as I have already shown, that the managers have not reckoned their anticipated charitable contributions among their assured assets, and in place of offering on certain terms a certain annuity which these terms fairly earn, together with the reasonable hope that a substantial addition may be made to the annuity out of the contributions referred to, have not reduced their premiums pro rata, or, in other words, do not saddle the Fund with the legal responsibility for liabilities which the unaided Fund could never meet. And then he goes on as follows: "We can only suppose that it is done in order that hereafter the managers of the Fund may have the opportunity of saying, 'see how much our performance has gone beyond our liability how it has even exceeded your most extravagant expectations. Such things are done every day by competitive insurance companies of various kinds. It is quite a trick of the trade to take a surplus pound by way of premium, and restore fifteen shillings of it to its delighted owner by way of bonus. The artifice is highly popular, and makes a splendid advertisement. It is mere finesse, but a section of the public account it finance." You see he actually charges Lord Rothschild, and Messrs. Hambro, Gibbs, and J. Morgan, whose representatives are on the Council, and who themselves have full cognisance of all the proceedings of the Association, and the other less important members of the Council, with the meanest form of dishonesty towards those whom it is their sole object to benefit, and all for doing what any clear-headed man of business must see it was their duty to do.

I have not hitherto said anything with regard to the sickness fund, because, although payment in sickness forms a part of the general scheme of the Pension Fund, it is quite distinct as a matter of business and in principle from the granting of annuities. On this branch of the subject the writer is specially eloquent; he inveighs against the limitations and restrictions with which the granting of policies for sickness is surrounded; and, by process of a confused jumble of arguments which it is not very easy to follow, arrives at the apparent conclusion that it is the duty of the Fund to enter into such contracts with no restrictions at all, merely because nurses are more liable to ill-health than others; and suggests that because it has not committed any such suicidal folly, the prospectus (which he thinks was incautiously made public) should be withdrawn, and a revised scheme should be prepared, "in consultation with those who know what nurses want, and can not only conjure with figures, but also understand the flesh-and-blood facts of the case." It would be an utter waste of time to treat such stuff as this seriously. It may impose upon some nurses and upon persons whose philanthropic impulses swamp their discretion. But those who have any practical knowledge of the matter, or who choose to think, must know that there is no more difficult business to conduct than that of a sick fund, with a view even to solvency alone, that there is none which so exposes itself to fraudulent claims, and that for this and other reasons very few indeed of the immense number of societies transacting such business alone are in a satisfactory condition. With the most earnest desire on the part of the managers of the Pension Fund to deal in this matter liberally with their clients, they dare not commit themselves to that imprudence and folly which the reviewer proclaims as liberality and wisdom. But in this, as in the other questions at issue between the reviewers and the managers of the Pension Fund, the decision must rest with experts, and not with amateurs; and the experts speak with no uncertain voice.

In concluding these criticisms on the *Lancet's* critic, I will call attention to one or two noteworthy facts. In the first place, although professing to admire the liberality of the gentlemen who floated the Fund, and (except for their dishonesty) the good intentions of those who are conducting it, he cannot discover a single item in the prospectus that merits his approval; it is nothing but a series of blunders from beginning to end. In the second place, he thinks that there are still hopes for the Fund if only a revised scheme be prepared, as he suggests, in consultation with those who know what nurses want. But he nowhere ventures to give the least direct assistance towards the solution of the problem. And here, no doubt, he is well advised. It is one thing to destroy, it is quite another thing to construct. He has held a brief for the prosecution, and having no case has adopted the well-known legal expedient. But he has not dared, and I venture to think never will dare, to formulate an alternative scheme of his own. So far as I can deduce any opinions he may have from his somewhat incoherent and contradictory criticisms, they seem to be—first, that he would eliminate the charitable element, so that nurses should only obtain what their subscriptions earn for them; next, that he would reduce the premiums below those which the fund demands and which a ctuaries declare to be necessary to insure safety; and lastly, that he would grant sick pay on easy terms and without restrictions of any kind to all applicants. A virtuous and attractive scheme, truly! But God help the poor women who should be tempted to invest their earnings on such terms.

Well, ladies and gentlemen, the Pension Fund may succeed in accomplishing the object of its promoters, or it may fail. The issue depends mainly on two things: Firstly, whether nurses can afford or are able to make present sacrifices for the prospective benefits which the Fund offers; secondly, whether the friends of nursing and of nurses will give adequate assistance in the matter. It must be admitted that (apart from the eleemosynary element) the scheme is not *primâ facie* specially attractive; but it is impossible that any strictly business-like arrangement of the kind can in this respect be rendered attractive. The collective repayments to assurers cannot exceed the collective subscriptions of the assurers, *plus* the interest which their subscriptions earn, and must be less than these latter sums by the cost of management. All that the managers of assurance companies can do to make their business attractive is, first, to establish their business on a sound financial basis; second, to demand of their clients the lowest terms compatible with security; third, to administer judiciously the funds entrusted to their keeping; and, lastly, to minimise the expenses of management. As to the financial basis of the present undertaking I need say nothing. It has never been impugned. That the moneys entrusted to it will be judiciously and honestly dealt with in the interest of the assurers is altogether indisputable. And for various reasons which have already been adverted to the expenses of management will be maintained at a far lower rate than that of any other assurance company in existence. The only item needing discussion is that concerning the terms of admission for assurers. I have said that these should be as low as is compatible with security of return. But this is a matter which must be determined by investigation and experience, and demands, on the part of a young office doing a new kind of business great caution. If the premium were made too low, it would mean the insolvency of the concern sooner or later, the probable payment in full to the early annuitants, but disappointment or total loss for the remainder. If they were made too high, the subscriptions immediately payable would be such as to deter some at any rate who would have been willing to subscribe on lower terms. At the same time, it is important to bear in mind that in this case there would be no real loss to the insurer, for (still disregarding the charitable element) the excess of payment in the beginning would all be made up to her in the course of time, out of the accumulating funds, in the form either of a gradual diminution in the premiums or of an enhanced annuity. It is obvious, therefore, that if a company errs at all, it is bound to err on the side of safety, and that its preimum charges should be on a somewhat higher scale than that calculated merely to make ends meet. It is not a trick of the assurance business to demand higher premiums than are absolutely necessary, in order that on returning the excess of payment in the form of bonuses the company may boast of the successful management of its business. The principle is a legitimate one, and is acted upon openly by all companies; which issue two kinds of policy, the one at a lower scale of premium which admits of no variation, the other on a higher scale of premium which includes participation in the profits. The policies of the Pension Fund are all of the latter kind. I think it will be readily understood, from what I have said, that it is impossible that the strictly business part of the Pension Fund can ever be made much more attractive than it is. A little diminution in the premium rate might possibly be made with safety even now, and may be anticipated in the early future. But those who find it difficult to pay the present charges will practically find equal difficulty in paying the reduced charges. There is every hope, however, that the Pension Fund will be able to give a larger return for the subscriptions paid to it than any assurance company can afford. Not only are its terms lower than those hitherto demanded in respect of Government annuities (which are not participatory), but Government at the present moment (in consequence of the reduction of the rate of interest on Consols) is entering into no fresh contracts pending the reconstruction of their tables in a direction less favourable to the public than their old tables.

I believe that there are many nurses who are willing to contract with the Pension Fund on the terms offered to them. But I must confess that I base my hopes of its success upon the association of the eleemosynary element with the strictly commercial part of the undertaking. There are two chief ways in which philanthropists may be of service to it. In the first place, benevolent-minded persons may subscribe by donations or by annual subscriptions towards the Bonus Fund; in the second place the managers of institutions employing nurses may pay in part or wholly their nurses premiums. By the former method the Bonus Fund will become augmented, and, as is hoped, the pensions payable to nurses will become larger than are due to them merely on account of their subscriptions. By the latter method certain nurses will be directly relieved to a greater or less extent of the annual subscriptions, which it is admitted must be a serious drain upon their too slender resources.

Such, in plain language, and without embellishment, are the advantages the Pension Fund offers to nurses. Some of the details of the scheme may admit of amendment, and the Council of the Fund is quite ready to give full consideration to all reasonable suggestions. But in its main features it must continue to be what its originators have made it. It can only be rendered more attractive than it is by expanding the eleemosynary element; an element the introduction of which, we are told, has already offended and alienated many ladies.

I trust, in the interests of their profession, that those nurses, who have permitted such feelings to influence their conduct, will reconsider this matter. The term "charity," at any rate in its offensive sense, is not applicable to the aid which the public is invited to give towards the objects of the Pension Fund. The position and services of nurses are unique; nurses are public benefactors, who, in the nature of things, can expect but little direct return for their services. The contributions which the public are invited to give are in recognition of such services, received or in prospect, are no more charity than are the pensions awarded to eminent individuals for distinguished services. I confess I should be grieved if the Pension Fund should prove a failure, for it has been launched under specially favourable conditions. And if it fails, I see no prospect of success for any similar philanthropic undertaking. I believe it will succeed.

### The National Pension Fund for Nurses.

All Communications should be addressed The Editor, The Lodge, Porchester Square, W.

### GRATEFUL AND APPRECIATIVE.

THE Secretary asks us to publish the following letter from an old nurse who has invested her savings of over £200 in the Fund:

I THANK you much for your kindness in answering my last, and for all the trouble you have taken on my account I feel truly grateful to the Council. My mind feels relief in its being where it is, although before it was perfectly safe it was lying dormant, which was a pity. The conditions you mention of course I should be glad to avail myself of, but I sincerely hope matters will not require such to be. It would indeed be sad. The whole thing has not yet become known, and perhaps may not be thoroughly appreciated in the present generation. This we find to be the case. I often feel angry and sorry to read this and that, cruel slurs on persons and things, but the less notice taken of petty, jealous scandal the better. Some people are so annoyed that another began a work which had not entered their mind to do; now they feel like the fox in the fable, and can scarcely contain themselves. No doubt as much injury as possible will be done so as to keep people from joining, but I hope and sincerely believe that all the spite and disagreeableness will be lived down after a time. A NURSE.

### OFFENDED AND ALIENATED.

WE have received the following letter from Miss C. J. Wood, formerly matron of the Hospital for Sick Children, Great Ormond Street, and now secretary to the British Nurses' Association. We publish it because it shows what a just and kindly appreciation Miss Wood and those who share her views have of the Pension Fund:—

I have seen in a contemporary that the National Pension Fund (for Nurses) is also open to the male salaried officials of hospitals. As a nurse I feel that a great honour has been done to our profession, as now we are all one. In hospitals in past days, unfortunately there have often been dissensions—one department in jealous competition with the other; but as the National Pension Fund has, with its keen eye, discerned that secretaries, dispensers, clerks, porters, etc., are all members of the nursing profession, such squabbles may be relegated to the past, because the various heterogeneous elements of the profession will now group themselves harmoniously under the recognised head of the nursing department in the various hospitals. What a proud distinction thus to hold out the olive-branch of peace! Furthermore, I see in this contemporary that the nurse is to pay £2 10s. per annum for her annuity, and the male subscriber is to pay £2 2s. for his annuity per annum. Thus again does the National Pension Fund (for Nurses) prove the friend of nurses, for this is nothing less than a hint to the managers of hospitals to re-adjust the salaries, otherwise it must be a misprint on the part of this contemporary or a miscalculation on the part of the actuary. I trust that first the "various elements of the nursing profession" may meet their managers in friendly conclave and point out to them the flood of light that has been thus thrown upon their relative positions, and then, with their re-adjusted salaries in their hands, they will flock to the office of the National Pension Fund (for Nurses) and subscribe for a pension.

A NURSE.

### A PITIFUL STORY.

MRS. L. MONTAGUE, Penton, Crediton, sends us the following:—

"With regard to the National Pension Fund, I think the following story, taken from one of Mr. G. R. Sims's articles on 'Life Dramas of the London Poor,' in *Cassell's Saturday Journal*, speaks for itself: 'For one old lady's story I must, however, endeavour to find room. She came in shivering out of the cold (to the casual ward) and answered the regulation questions. Her name was Venning; she was the widow of a veterinary surgeon. Her husband died suddenly and left her nothing. Then she supported herself by needlework, being very clever and having obtained the certificate of South Kensington as a high art needlewoman; and, in addition to this, she had walked Queen Charlotte's Hospital and obtained a certificate as nurse. But ill-health and increasing age (she was seventy-one) prevented her nursing; and a finger, which gathered terribly, especially in the winter, stopped her doing needlework, and so she was only able to do a little now and then. When she earned a little she lived at a common lodging-house; when she couldn't work she came to the casual ward.'"

### TEAR AND WEAR.

EVERYONE is familiar with the fact that it is not mental work which kills a person, provided this mental work be accompanied by due periods of rest or repose, or change of mental activity. One says it is anxiety or worry that kills. What does mental worry mean when interpreted on a physical basis? It means that there is a continuous tear and wear of the tissues of the brain itself, and consequent liberation of highly-evolved and specialised forms of energy from the brain; that the brain has not a sufficient interval of time to restore its structure, and so there is a continual degradation in the molecular constitution of the brain itself thereby produced. Why, again, is sleeplessness of a serious symptom in the overworked man? Sleeplessness means absence of rest of the brain, absence of that process of repair which is necessary to the healthy action and continued existence of one's mental stability.

FOR all who are occupied with severe and continuous mechanical labour, a mixed diet, of which cereals and legumes form a large proportion, and meat, fish, eggs, and milk form a moderate but constant proportion, is more nutritious and wholesome than almost entirely animal food. For those whose labour is chiefly mental, and whose muscular exercise is inconsiderable, still less of concentrated nitrogenous food is desirable. A liberal supply of cereals and legumes, with fish and flesh in its lighter forms, will better sustain such activity than large portions of butcher's meat.—*Sir Henry Thompson, F.R.C.S.*

### Everybody's Opinion.

### THE ROUGH SIDE.

"E. L." wishes some one would speak of "the rough side of hospital life," bad living, short time for meals, etc. May I make a statement without being considered conceited, or setting myself up as a very extra good contriver? I am lady superintendent of a small hospital (50 beds), and I think my nurses cannot complain of the roughness of which "E. L." writes. I allow half-an-hour for breakfast, dinner, tea, and supper. Lunch we have no time for, except in very cold weather, when I often make them a cup of tea myself. For breakfast, eggs, bacon, sausages, or fish; dinner, meat, pudding or tart, with often two vegetables; bread-and-butter for tea, constantly varied with marmalade or cake; supper, meat, eggs, cheese, tea, coffee, or cocoa; for as my staff consists of "pledged abstainers," they are, I think, entitled to a change of "drink." On Sunday there is always some little extra. On birthdays a cake, and, if possible, a half-day off. Life in a hospital is at best a hard one. Nurses obtain very little consideration, and from my own experience as a "pro." I know meals are very rough, and not always ready; and why should not a good nurse (who is expected to be gentle and refined in manner) have all as nice and comfortable as it can be made, with all due consideration for the hospital funds? I find that with a little trouble and contrivance meals for nurses and patients can be a good deal varied from the "always mutton and beef;" and with all the change I have not found my monthly bills increase. A. E. S.

### WORKHOUSE NURSES.

We have received the following letter from Miss Wilson, Honorary Secretary of the Workhouse Infirmary Nursing Association:

I have just read your notice of our Association. Thanks for inserting it, as publicity is very useful. Though I find a good deal of difficulty in getting the exact sort of nurses I want, our Association does flourish; and I count more on quiet, steady work by each individual member than on any other form of effort. I do not think, however, from my nine years' experience in selecting nurses, that the "material" I have to choose from has improved. This seems odd when so many more are trained; but amongst the nurses who offer themselves now we have even more "sifting" than formerly. Some of our best nurses were our earliest. Of course in our own probationers it is different, but I am referring to those already trained who come anxious to join us. Of course some blame must fall on the public for their ignorance of what a workhouse infirmary nurse ought to be. The strangest people are sent to us—deaf, half-lame, totally inefficient as nurses, unsatisfactory as women. Sometimes we are asked to take or "reform" nurses who have the habit of taking opium or some other grave vice! I often feel a little indignant at the sort of nurses even some matrons are willing and axious to place in charge of the poor sick inmates of a workhouse infirmary. I can only fancy they do not realise why only the best nurses are wanted for such work. The second best quickly become discouraged under the difficulties of a county union, and degenerate into indifferent workers; there is so little real supervision that this is but a natural consequence. Our Association being specially formed to raise the standard of infirmary nursing, we try to appoint only those women who will make of difficulties opportunities for gradual progress and achievement. As you point out in your paper, such women are rare, but they are to be found, as, I am glad to say, we have experienced. The reform in having a higher class of officials over the workhouses will do much to change the difficulties of the present situation, especially for nurses. The guardians at Oxford hope to set a good example shortly by appointing a lady as matron at their workhouse, and I have been able to recommend several to them. I can only hope their example will be followed by other Unions. The benefit to the able-bodied women, children, the sick, and the officials would be incalculable, if having a refined woman over them. Of course such a lady must understand the care of linen, laundry work, cutting-out, and be, in fact, generally well trained in domestic matters. Shortly our larger workhouses may be open to train in this branch of work, for training is as useful here as in nursing. Perhaps if your readers care for our Association they would like to have a notice of our annual gathering of nurses. J. Wilson.

### NURSE TRAINING SCHOOLS IN AMERICA.

We have received the following communication from the superintendent of an American nurse-training school. The fault it deals with is too common in this country, and here, as on the other side of the Atlantic, efforts are being made to remedy it:—

The attention of several of the larger training schools for nurses in America is becoming aroused to the probability of a serious injury being inflicted on them by the sudden growth of the so-called "nurse school" which is to be found attached to every hospital of twelve beds or so. Generally speaking, the medical men on the staff of these hospitals are opposed to the cultivation of the nurse school And this not, at the present time of grace, because they object to the care of their patients by skilled nurses, but because in a hospital which can afford but few they desire that each individual should be competent to her business. The management, on the contrary, sees a certain pecuniary benefit to be derived from obtaining the services of an untrained woman at a third of the price they would offer to skilled labour, and consider they are justified in making such an arrangement by giving, instead of training, what is known as the "run of the hospital." Women who desire to become nurses are not always acquainted with the superior advantages to be derived from the large training schools, and are easily diverted from them by some very trivial attraction. Much valuable nurse material is wanted in this way, and benefits are bestowed on those not always best able to appreciate them. It is proposed later in the year that a conference of superintendents of training schools should be held to discuss this and kindred subjects. It may be mentioned that in the city of Philadelphia alone, while there are six large hospitals with prosperous and well-managed training schools, there are at least seven which have no right to an existence at all. In many cases a diploma is given by these schools, which is ridiculously over-valued. Philadelphia.

### THE HYSTERICAL STYLE.

I have met with a weekly paper which gives utterance to most noble views with regard to nurses, yet by its style fosters in them the very habits which nurses should most avoid. In the first number there was scarcely a page which did not contain italics and marks of exclamation, and now I see its language is so strong that small capitals have to be called into requisition to aid the poor overburdened italics. Now, a nurse, of all women, should be the last to join the shrieking sisterhood, and should be careful to always preserve a calm, self-contained manner; therefore the less she reads of this hysterical journal the better. Nurse Black, Glasgow.

### THE PROBATIONER'S PROTEST.

In the hospital I entered as paying probationer I had charge of thirteen beds. My duties were to make my patients' beds, sweep the floor, polish a long ward table, wash all the crockery used in the ward (during the day), scour a sink, polish taps, and minister to the wants and comforts of all my patients. On complaining to the matron of having so much menial work, I was told it was part of my training. Need I mention that after a month of such wear and tear my health gave way? Who can remedy this evil—the matron or doctors? I feel very strongly about the rough side of hospital life, believing it to be an unnecessary evil, which can be stamped out by a little more consideration on the part of those in authority. To the probationer, who is often so tired—too tired even to go out when "off duty"—hours of such work are given. I fear there is a tendency in hospital life to forget that the bravest workers grow weary at times. Let one and all nurses watch for the opportunity of giving that encouragement to a fellow-worker, even the probationer.

SISTER NINA LEE.

### Anecdotes of Personal Adventure.

READING the experiences of my sister nurses reminds me vividly of a little embarrassment I experienced a day or two after my arrival at my first case. My patient was an elderly lady, and mentally afflicted. One of her sons, who had been quite recently married, called upon his mother and brought with him his bride; but my patient's idea was that I was the bride, and was much distressed to see us so very formal in our greetings, and immediately besought us to demonstrate more affection by embracing each other. I made some excuse, glad to escape from the drawing-room and visitors, and so set all at ease. *S. E. A.*

SOME years ago another nurse and myself were engaged in nursing an old lady, who lived in a lonely house near a large town. The household consisted of two ladies and two servants. I was on day duty, and slept at night in a small room near the patient's. One night, soon after twelve p.m., I was awakened by the night nurse bidding me, in a hoarse whisper, get up at once, something was the matter. I scrambled hastily into my dressing-gown and went on the landing, where I found the night nurse standing holding a lighted candle. She looked frightened, and told me she had heard strange sounds proceeding from the lower regions, and suspected that burglars were trying to get into the house. We listened a few minutes and then heard a great noise like a window being smashed in; a current of cold air rushed up the stairs and put the candle out. I was panic-stricken, and uttered a piercing scream which aroused the whole household. The cook was first to arrive on the scene; she called loudly to some imaginary person to "bring the gun at once," hoping to frighten off the would-be burglars. We waited and watched three or four hours; all was quiet, and we hoped they had gone; still no one liked to venture downstairs. It was beginning to get light. We opened a window and were delighted to see a policeman outside. We told him our fears. He said that the front door had been left on the chain, and he had rattled it to arouse someone to go down and fasten it. We were all much relieved to hear that our supposed burglars were a myth. The patient laughed heartily at our mistake, and, I am glad to say, suffered no ill-effects from our false alarm. *Aidyl.*

A CERTAIN probationer in a well-known hospital did not consider the patients were sufficiently impressed with the fact that she was a lady, and on one occasion took an opportunity of informing them of the fact, saying she had never been used to work, etc. When she had finished, a man in a bed near her quietly remarked, "Ah! there's a many ladies as nurses here, but you're the first that's had to tell us so!"

THAT which creates a people is domestic life. The loss of it degrades a people to a horde. The authority and obedience, the duties and the affections, the charities and the chastities of home are the mightiest and purest influences in the formation of human life. A good home is the highest and best school; it forms and perpetuates the character of a nation.

### Original Articles.

### NURSING THE INSANE.

NURSES, or as they are more commonly called, attendants for the insane, are a class of women whose training is too often neglected, and not brought to a proper pitch of perfection. They are left to learn their profession by experience, and it is seldom that a doctor thinks of lecturing to them, or explaining fully the treatment he wants carried out. Luckily there are a few good books on the subject, such as the small handbook by Dr. Charles K. Mills, but these are not well known, and asylum nurses who study at all are apt to attack huge volumes on insanity of a purely medical nature, which are of little use except to doctors. In some of the large asylums for insane paupers there are two thousand patients. These include all species of lunatics, from the raving maniac down to the helpless imbecile. The nurses are chiefly recruited from the domestic-service class, and get £15 the first year, afterwards rising to £25 a-year. They are on duty from six in the morning till eight at night, with half-a-day off duty once a week and a whole day every month. In their second year they get a fortnight's holiday in the summer, and the same in succeeding years. As a rule they like their work and stick to it, and on leaving one asylum go into another. Of course in a pauper asylum the actual work demanded of a nurse is very light; she has chiefly to superintend the cleaning, &c., of her department by the patients under her care. In the laundry, the kitchens, the garden, and everywhere, the patients who are capable are permitted and encouraged to work. Of course an asylum nurse has to run the risk of being knocked about; she is almost sure to lose a few handfuls of hair and get some bruises if she is long amongst the insane; but more severe injuries need not be dreaded. The arrangements are so perfect that at the first symptom of violence a patient is rapidly secured and shut in the padded room; here her struggles can hurt neither herself nor others, and her cries are heard by none. The nurse in charge of the department devoted to violent patients is always an experienced woman, and one of great bodily strength. Her post is not such an anxious or exciting one as the outside public might suppose, and she, together with her sister nurses, will complain of the monotony of asylum life. In the infirmary the nurses are untrained so far as hospital experience goes; though the average number of deaths may be as high as twenty in a month. Pauper lunatics are often under-sized, weakly, and lacking in vitality; they are seldom long ill; they have no stamina to stand against any disease which attacks them, and death comes very quickly. Nursing the insane in private asylums or doctors' houses is well-paid work, and often very pleasant. Fifty pounds is a common salary to a woman so employed, if she is thoroughly experienced and generally capable. A lunatic soon learns who fears her, and likes to work on such fears, so that presence of mind and courage are indispensable in their attendants. Nurses have died from injuries received from their patients, as hospital nurses have died from infection secured in the same way; and perhaps care of the insane in a private house is more risky than in an asylum, for after all there is safety in numbers, and patients will protect a nurse they have learned to like. A clever nurse should always know, from various signs, when a patient is approaching an attack of mania, and take precautions. She should also know which particular form of insanity each patient is suffering from, whether it be melancholia, epilepsy, the insanity which is associated with general paralysis, etc. If possible, she should try and be sympathetic even with their manias, and by a judicious system of work and recreation try and keep the attention distracted from the dangerous point. Attendants on the insane have to cultivate to a very high degree the powers of observation, self-control, and patience, which all nursing demands, and their work is often most wearisome and depressing. They have our sympathy.

### Appointments.

MRS. BRUNCKER has been appointed matron of the sanatorium in connexion with Wellington College. Mrs. Bruncker trained at the Middlesex Hospital, and was afterwards a sister there. As she is a widow with an only child, the lucrative post she has now secured in a beautiful and healthy locality is a subject of congratulation.

### Examination Questions.

The following questions were given to probationers in a provincial hospital:—

(1) Translate the following terms and give the abbreviations:

| | |
|---|---|
| Alternâ-nocte. | Si opus sit. |
| Ante cibum. | Sumendum. |
| Bis die. | Statim. |
| Hâc nocte. | Ter die. |
| Horæ somni. | Parti dolenti. |
| Mane. | Ad libitum. |
| Nocte. | Cras-mane. |
| Nocte maneque. | Quâquâ hora. |
| Omni mane. | Utendum. |
| Post cibum. | Parti affectæ applicandum. |

(2) When should you give a purgative medicine—on an empty or full stomach?

(3) State the best way of giving oils. When should cod-liver oil be given?

(4) When should arsenic, iron, and quinine be given—before or after food?

(5) Read the following prescriptions:

Magnesiæ Sulphatis ℥jss.

Extracti Glycyrrhiza ʒij.

Tincturæ Sennæ ℥j.

Tincturæ Cardamomi Compositæ ʒiv.

Infusi Sennæ ad ℥viij.

Misce Fiat Mistura: Sumat Cochlearia duo magna quartis horis ad effectum.

℞ Magnesiæ Sulphatis ℥ij.
Infusi Rosæ Acidi ℥viij.
Misce. Fiat Mistura: Sumat Cochlearia duo magna ad effectum.

### Notes and Queries.

TO CORRESPONDENTS.—1. Questions may be written on post-cards. 2. Advertisements in disguise are inadmissible. 3. In answering a query please quote the number. 4. If a private answer is desired, a stamped addressed envelope must be forwarded.

18. I should be obliged if you can tell me through THE HOSPITAL the name or names of some good books for reference and study (medical) for a nurse engaged in private nursing, and if it is possible to obtain then second-hand. NURSE E.—Application may be made to the Secretary, Hospitals Association, Norfolk House, Norfolk Street, Strand, W.C.

19. I should be glad to know of any information of St. Katherine's Hospital, Regent's Street; and how has the Queen given the Jubilee money for nurses nursing among the sick poor. How can one profit by it doing so? To whom must one apply to get on the staff, and any rules? or how it is worked with St. Katherine's? Anyone answering it will greatly oblige yours, &c., A.

20. Is £4 per head for stimulants during ten years in a small hospital not considered a large amount? What is the average amount? MATRON OF A COTTAGE HOSPITAL.

21. *M. H. Lawrence* would be glad to know if he can obtain THE HOSPITAL in Canada.—The best way to get THE HOSPITAL in Canada is to order it from The International News Company, New York, or through a leading newsagent there. It can be had direct by post on payment of 6s. 6d. per annum in advance.

22. Could you inform me through the medium of your paper if there is a District Nursing Association in the neighbourhood of Pimlico, S.W.; or in Pimlico or Clapham?—The London, Clapham, and Brixton Nurses' Association, 210, Clapham Road, S.W.

23. I should be glad to know where "papier goudronne," mentioned in "Aids to Nursing," can be obtained.—A COUNTRY MIDWIFE.

24. Will you kindly inform Nurse J. through the medium of THE HOSPITAL "Notes and Queries" if there are any vacancies in or about Dublin for hospital-trained Nurses (certificated.)

25. Will you kindly tell me through your paper where I should write for information concerning the Metropolitan Nursing Association and the East London Nursing Society.—SILCOTE. [Apply to Secretary in each case at the institutions, (1) 23, Bloomsbury Square, W.C.; (2) 49, Philpot Street, Commercial Road, E.

### Answers.

*Miss Islip.*—We have forwarded the letter with pleasure to the Hon. Secretary, Mr. H. W. Large, Middlesex, and hope you may secure what you wish.

24. There is no vacancy for a trained Hospital Nurse at present in any of hospitals in Dublin or the neighbourhood.

*Mrs. Denison.*—Your letter has been forwarded to Miss Wilson, Hon. Secretary of the Workhouse Nursing Association.

### Vacancies.

The following vacancies are announced:—

### Matrons.

Worcester Infirmary.
Dewsbury General Infirmary; £50.

### Lady Superintendents.

Sheffield Nurses' Home; £70.
Queen's Hospital, Birmingham; £7

### Nurses.

Bradford Eye and Ear Hospital; £25.

Knighton Cottage Hospital; £25.

Royal Albert Hospital, Devonport; £25.

West Herts Infirmary; £22.

Miller Hospital, Greenwich; £22.

Weston-super-Mare Hospital; £25.

Hertford Infirmary; £20.

Central London Ophthalmic Hospital; £35.

Birkdale Nurses' Home.

Swansea Nursing Institute.

Children's Hospital, Sheffield.

Home Hospital, Fitzroy Square.

Kingston Home, Hull.

Burton-on-Trent Nursing Institution; several; £25 to £28.

Seamen's Hospital (late *Dreadnought*), Greenwich; £20.

Bristol Royal Infirmary; several; £25 to £28.

Nottingham and Notts Nursing Institution; several; £25 to £30.

Metropolitan Hospital, Kingsland Road; £20.

Workhouse Infirmary Nursing Association; several.

Glasgow Sick Poor and Private Nursing Association; several.

Bristol Royal Infirmary; several.

Bradford Eye and Ear Hospital; £25 to £30.

Brighton Borough Fever Hospital; £25 10s.

Swansea and South Wales Nursing Institute; two.

Birkdale Trained Nurses' Home, Southport.

Kingston Home, Baker Street, Hull.

### Probationers.

Bristol Hospital for Sick Children; three.

Brighton Borough Fever Hospital; £18 12s.

### Exchange and Mart.

UNDER this heading we propose to insert purely Exchange advertisements, at a charge of sixpence for eighteen words, or three insertions for one shilling It must be distinctly understood that ordinary advertisements will not be inserted here.

RENSHAWS MANUALS. — "Anatomy," Malgaine (12s. 6d.), "Elementary Botany, Dr. Silver (12s. 6d.), "Human Osteology" Ward (7s.) Would exchange either for Nurse's Surgical dressing case. What offer? Also Owen's "Materia Medica," "A Guide to the New Pharmacopœia" "First Book of Botany, Balfour. The last three for 5s.; or one guinea the lot. All in good condition. Address C., THE HOSPITAL Office, 38, Old Jewry, E.C.

